# Toxicity of Jegosaponins A and B from *Styrax japonica* Siebold et al. Zuccarini in Prostate Cancer Cells and Zebrafish Embryos Resulting from Increased Membrane Permeability

**DOI:** 10.3390/ijms22126354

**Published:** 2021-06-14

**Authors:** Moe Nishimura, Hiroyuki Fuchino, Kaoru Takayanagi, Hitomi Kawakami, Hiroko Nakayama, Nobuo Kawahara, Yasuhito Shimada

**Affiliations:** 1 Department of Integrative Pharmacology, Mie University Graduate School of Medicine, Tsu, Mie 514-8507, Japan; 318070@m.mie-u.ac.jp (M.N.); 316066@m.mie-u.ac.jp (K.T.); 2 Research Center for Medicinal Plant Resources, National Institutes of Biomedical Innovation, Health and Nutrition, Hachimandai, Tsukuba, Ibaraki 305-0843, Japan; fuchino@nibiohn.go.jp (H.F.); h-kawakami@nibiohn.go.jp (H.K.); kawahara@nibiohn.go.jp (N.K.); 3 Zebrafish Drug Screening Center, Mie University, Tsu 514-8507, Japan; nakayama@innov.mie-u.ac.jp; 4 Graduate School of Regional Innovation Studies, Mie University, Tsu, Mie 514-8507, Japan; 5 The Kochi Prefectural Makino Botanical Garden, Godaisan, Kochi 781-8125, Japan; 6 Department of Bioinformatics, Mie University Advanced Science Research Promotion Center, Tsu, Mie 514-8507, Japan

**Keywords:** screening, anti-cancer, toxicology

## Abstract

(1) Background: Screening of medicinal herbs is one of the most powerful approaches to identifying novel therapeutic molecules against many human diseases. To avoid potential harmful effects during medicinal use, toxicity testing is necessary in the early stages of drug discovery. The objective of this study was to identify the cytotoxic mechanisms of jegosaponin A and B from *Styrax japonica* Siebold et al. Zuccarini; (2) Methods: We screened Japanese medicinal herb extracts using PC-3 prostate cancer cells and found that a methanol extract isolated from the unripe fruit of *Styrax japonica* Siebold et al. Zuccarini (SJSZ) had an inhibitory effect on cell viability. We further performed fractionation assays with PC-3 cells and identified the bioactive compounds using LC/MS and NMR analysis. We clarified the toxic mechanisms of these compounds using PC-3 cells and zebrafish embryos; (3) Results: We identified two active molecules, jegosaponin A and jegosaponin B, in the inhibitory fractions of the methanol extract. These jegosaponins are toxic to zebrafish embryos during the early developmental stage. Jegosaponin A and B showed strong haemolytic activity in sheep defibrinated blood (EC_50_ = 2.1 μM, and 20.2 μM, respectively) and increased the cell membrane permeability in PC-3 cells and zebrafish embryos, which were identified using a membrane non-permeable DRAQ7, a fluorescent nucleus staining dye; (4) We identified the cytotoxic compounds jegosaponin A and B from SJSZ, which we showed to exhibit cell membrane disruptive properties using cell- and zebrafish-based testing.

## 1. Introduction

Constituents of natural products are an important source of medicine [1], and as their chemical diversity can provide the core scaffolds for future medicines, a number of promising drug candidates, currently in development or in the market, originate from natural products [2]. Drug discovery from plants has been ongoing since ancient times and is still undergoing various modified protocols, such as high-throughput screening and in vivo testing, including in zebrafish [3,4,5]. In addition, there is an increasing use of herbs as traditional and alternative medicines, and there is a strong demand for more scientific evidence to evaluate their efficacy and safety.

*Styrax japonica* Siebold et al. Zuccarini (SJSZ) is a shrub of the family Syracaceae, native to Japan, China, and Korea. The species also possesses high commercial, nutritional, and therapeutic values, and has long been used conventionally by many communities for treating oral and dental diseases and respiratory ailments [6]. Several bioactive molecules have been identified in SJSZ in previous studies. For example, norlignan and styraxlignolide have anti-complement activity [7] and styraxoside A has anti-inflammatory effects in cultured cells [8]. In addition, phytoglycoprotein (38 kDa) from SJSZ suppresses cell proliferation in human carcinomatous cells [9,10] and mice [11].

As major physiological processes and gene expression pathways are highly conserved in vertebrates, zebrafish (*Danio rerio*) have been an attractive model for toxicological testing denoted by the zebrafish embryo acute toxicity test (ZFET) and can serve as an alternative model to rodents [12,13]. The Organisation for Economic Co-operation and Development (OECD) recommends the use of the ZFET for in vivo assessment of the safety of chemicals [14]. In addition, zebrafish embryos, larvae, and juveniles have transparent body walls, which enables live imaging using tissue-specific proteins and fluorescent dyes. For example, we previously evaluated the neurological toxicity of fluorescent indoline derivatives in zebrafish embryos [15,16].

The objective of this study was to identify the cytotoxic mechanisms of jegosaponin A and B from *Styrax japonica* Siebold et al. Zuccarini. As a preliminary study, we performed cell-based screening of 756 Japanese herb extracts using human prostate cancer cells and thereby identified the cytotoxicity of the extracts from unripe fruit of SJSZ. In this study, we identified two bioactive constituents, jegosaponin A and jegosaponin B, as underlying this cytotoxicity, and clarified the mechanism of cytotoxicity in cultured cells and zebrafish embryos.

## 2. Results

### 2.1. Jegosaponins from Styrax japonica Siebold et al. Zuccarini (SJSZ) Suppresses Cell Viability in PC-3 Prostate Cancer Cells

**Figure 1:**
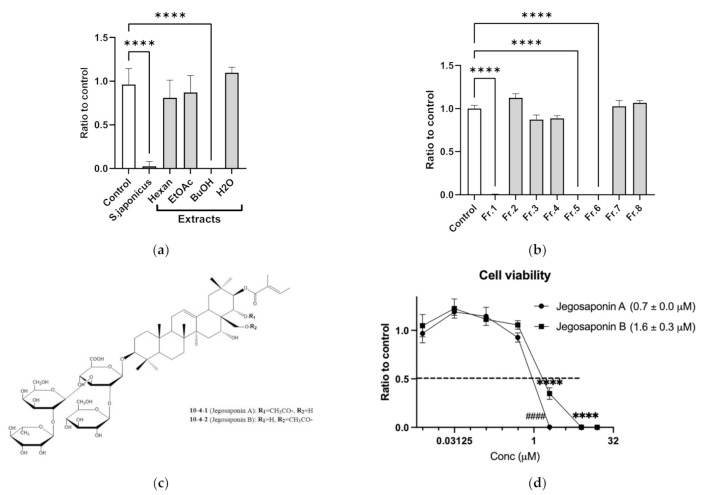
Jegosaponin A and B from unripe fruits of *Styrax japonica* Siebold et al. Zuccarini (SJSZ) suppress prostate cancer PC-3 cells. (**a**) BuOH extract of SJSZ suppresses PC-3 cell viability. *n* = 8, error bars indicate SD. **** *p* < 0.0001 compared to control (0.1% DMSO). (**b**) Fraction 1, 5 and 6 of BuOH extract of SJSZ suppresses cell viability in a dose-dependent manner. *n* = 8, error bars indicate SD. **** *p* < 0.0001 compared to control (0.1% DMSO). (**c**) Chemical structures of jegosaponin A and B. (**d**) Jegosaponin A and B suppress PC-3 cell viability after 48 h treatment. *n* = 8, error bars indicate SD. **** *p* < 0.0001 compared to control (0 μM jegosaponin A). #### *p* < 0.0001 vs. control (0 μM jegosaponin B).

Cell-based screening identified that methanol extracts of SJSZ (10 μg/mL) strongly suppressed PC-3 cell viability. To determine the responsible constituents, we performed serial extraction (*n*-hexane, ethyl acetate, and *n*-butanol) and subsequent cell viability assays, and determined that the *n*-butanol extract strongly suppressed PC-3 cell viability (10 μg/mL each; Figure 1a). We further performed fractionation of the *n*-butanol extract, and again performed subsequent cell viability assays with the products, and identified that fractions 1, 5, and 6 suppressed PC-3 cell viability (Figure 1b). By LC/MS and NMR analyses, we found these fractions to contain jegosaponin A and jegosaponin B (Figure 1c), which suppressed PC-3 cell viabilities at lethal concentration 50 (LC_50_) = 0.7 ± 0.0 μM and 1.6 ± 0.3 μM, respectively (Figure 1d). In addition, we confirmed that these jegosaponins also suppressed the viability of other human prostate cancer cell lines, namely DU145 and LNCaP (Figure S1).

### 2.2. Acute Toxicity of Jegosaponins in Zebrafish Embryos

**Figure 2:**
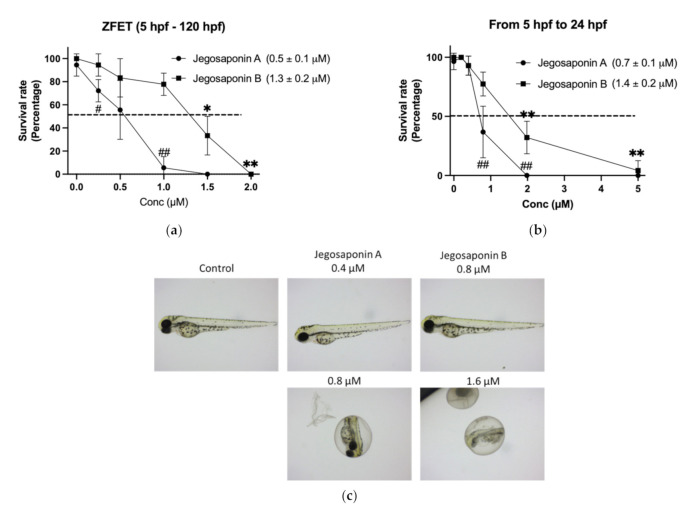
Zebrafish embryo acute toxicity test of jegosaponin A and B. (**a**) Blastula (5 hpf) embryos were exposed to jegosaponin A and B for 120 hpf. *n* = 3, * *p* < 0.05 and ** *p* < 0.01 vs. control (0 μM jegosaponin A); ## *p* < 0.01 vs. control (0 μM jegosaponin B) (**b**) Survival analysis on 24 hpf with jegosaponins exposure. * *p* < 0.05 and ** *p* < 0.01 vs. control (0 μM jegosaponin A); ## *p* < 0.01 vs. control (0 μM jegosaponin B). (**c**) Representative images of (**a**).

To evaluate the in vivo toxicity of the jegosaponins, we performed a zebrafish embryo acute toxicity test (ZFET) according to the OECD guidelines [14]. After 120 h administration of jegosaponins from 5 h-post fertilization (hpf) embryos, LC_50_ of jegosaponin A and B was 0.5 ± 0.1 μM and 1.3 ± 0.2 μM, respectively (Figure 2a). In the time-course experiments, we found that the toxicity of the jegosaponins 24 h after administration was almost the same as that at 120 h (Figure 2b; LC_50_ of jegosaponin A and B was 0.7 ± 0.1 μM, and 1.4 ± 0.2 μM, respectively). This suggests that the toxicities of jegosaponins were exerted in a relatively short time. The typical phenotypes induced by jegosaponin A and B are represented in Figure 2c.

### 2.3. Cytotoxic Mechanisms of Jegosponin A and B

**Figure 3:**
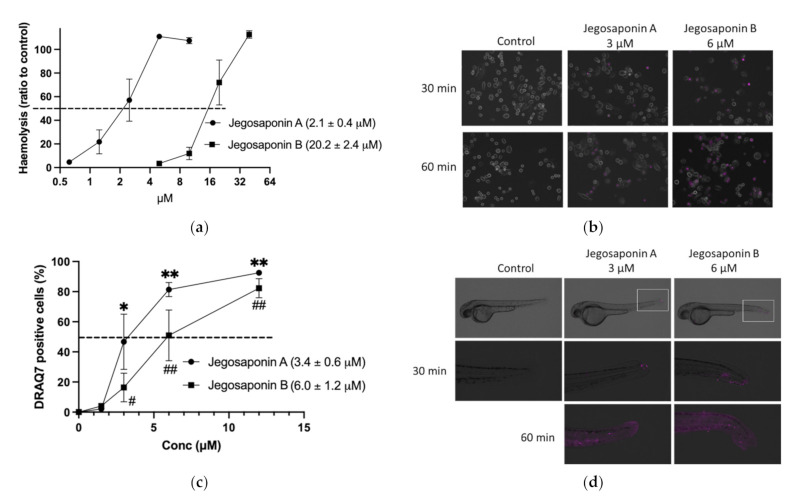
Jegosaponin A and B increase cell membrane permeability. (**a**) Haemolysis of RBCs by jegosaponin A and B. *n* = 3, error bars indicate SD. (**b**) Increase in cell membrane permeability of PC-3 cells treated with jegosaponin A and B. Purple indicates membrane impermeable DRAQ7-stained nucleus. (**c**) Quantitative analysis of (**b**). *n* = 3, error bars indicate SD. * *p* < 0.05 and ** *p* < 0.01 vs. control (0 μM jegosaponin A); # *p* < 0.05 and ## *p* < 0.01 vs. control (0 μM jegosaponin B). (**d**) Increase of cell membrane permeability of 48 hpf zebrafish by jegosaponin A and B. Purple indicates membrane impermeable DRAQ7-stained nucleus.

As saponins are well-known non-ionic surfactants, which can induce permeabilization of the cell membrane, we measured the haemolytic activities of jegosaponin A and B in sheep red blood cells. As shown in Figure 3a, the concentrations of the 50% haemolytic dosage (HD_50_) in jegosaponin A and B were 2.1 ± 0.4 μM, and 20.2 ± 2.4 μM, respectively.

To evaluate the jegosaponin-induced increase in cell membrane permeability in PC-3 cells, we administered DRAQ7, a cell-impermeable DNA staining far-red fluorescent dye, with jegosaponin A and B (Figure 3b; purple indicates DRAQ7-stained nucleus). The nucleus DNA was stained in a time-dependent manner after treatment with either jegosaponin (EC_50_ of jegosaponin A, 3.4 ± 0.6 μM; jegosaponin B, 6.0 ± 1.2 μM; Figure 3c), indicating that these jegosaponins disrupt the cell membrane to induce cytotoxicity. The negative control (0.1% DMSO) did not allow DRAQ7 to stain the nucleus.

Next, we performed DRAQ7 treatment on zebrafish larvae to validate whether jegosaponins could increase cell permeability under in vivo conditions. As shown in Figure 3d, after 30 min of exposure, jegosaponin A and B apparently increased DRAQ7 signals (purple) mainly in the tail regions, indicating that the jegosaponins increased cell membrane permeability in zebrafish (48 hpf) skin, allowing DRAQ7 incorporation into the nuclear DNA. In addition, the high concentrations of jegosaponin A and B (6 μM, and 12 μM, respectively) killed all embryos for 60 min.

## 3. Discussion

Saponins are glycosides and triterpene glycosides in plants and are considered useful in the treatment of human diseases, including cancer. Yoshikawa et al. previously identified jegosaponin A, B, C, and D from fresh fruits of SJSZ and determined their structures [17], but their biological activities have not yet been characterised. In this study, we found that SJSZ extract contains anti-cancer constituents, which act against human prostate cancer cells, as subsequently identified them as two jegosaponins, jegosaponin A and B. We further found that the cytotoxic effects of jegosaponin A and B were fast acting in both cultured cells and zebrafish embryos, implying that the main underlying mechanisms involve direct toxicity, probably by inducing the destruction of the cell membrane, rather than by ROS accumulation or alteration of gene expression profiles in other bioactive constituents in SJSZ, such as 38 kDa glycoprotein [9,11]. Therefore, their use as anticancer drugs is difficult. As some saponins are reported to have apoptotic properties in cultured cells [18], we measured the activities of caspase-3 and caspase-7, direct executers of apoptosis, after treatment with jegosaponin A and B, and found no apparent induction of their activities even at sub-IC_50_ concentrations (Figure S1).

In this study, we determined the HD_50_ and LC_50_ of jegosaponin A and B for the first time (Table 1). Although the chemical structures of jegosaponin A and B were quite similar (Figure 1d), all parameters determined in this study were higher in jegosaponin A than in jegosaponin B. Jegosaponin A and B are positional isomers of acetyl and hydroxyl groups, respectively. For the structure-activity relationship, as with other saponins, the triterpene moiety of both jegosaponin A and B is hydrophobic and the sugar moiety is hydrophilic, thus, they are considered to have sufficient surfactant activity. Jegosaponin B differs from jegosaponin A in that the acetyl group attached to the C-28 position (Figure 1c) has an axial configuration, so it is sterically repulsive to the tigloyl group attached to the C-21 position. In other words, the conformation of the bulky tigloyl group is expected to be very different between A and B, which is thought to be the reason for the difference in activity observed in this study. In fact, in jegosaponin B, the acetyl group and the tigloyl group are arranged in a 1,3-diaxial configuration, which is prone to steric repulsion, so it is likely that the tigloyl will face outward to mitigate repulsion, and the difference may be large.

In addition, jegosaponin A and B have an αβ-unsaturated carbonyl (Michael acceptor); therefore, if a strong nucleophilic reagent, such as the SH group is present in our assay system, Michael addition may occur irreversibly. For example, the SH group of the enzyme may attach to the Michael acceptor causing non-selective enzyme inhibition. To be honest, it is difficult to prove that this phenomenon did not occur in our assays. However, there are many natural products that have such substructures, such as sesquiterpenes and diterpenes from the Asteraceae family, but given that we were unable to identify any in our screening study, we believe that the effect is negligible in this assay.

Voutquenne et al. summarised the haemolytic activities of saponins based on their structures [19]. Compared to other saponins and sapogenins, the HD_50_ of jegosaponin A and B are quite low, especially that jegosaponin A (HD_50_ = 2.1 μM), which indicates the high toxicity of these molecules in mammals. The surfactant properties of saponins are generally used in a wide range of applications, including expectorants, detergents, and emulsification of fragrances and spices. In the tropics, they are also used to remove snails that serve as intermediate hosts to reduce schistosomiasis. Jegosaponin A and B have the strongest surfactant activity among the saponins and are expected to be used for these applications.

The ability of these jegosaponins to disrupt cell membranes was demonstrated in prostate cancer cells and zebrafish embryos (Figure 3). In zebrafish embryos, we found that the tail fin is quite sensitive to jegosaponin-induced membrane destruction, which allowed the incorporation of membrane impermeable DRAQ7 into the cells. For the first time, we visualised cell membrane permeabilisation in vivo, which could offer a tool for evaluating the toxicity of other detergents.

## 4. Materials and Methods

### 4.1. Instruments

LC/MS was performed using an Orbitrap Elite spectrometer (Thermo Fischer Scientific, Waltham, MA, USA) equipped with an electrospray ionisation (ESI) ion source. HPLC was conducted for purification on a Shimadzu LC-10ADvp series (Shimadzu, Tokyo, Japan). ^1^H and ^13^C-NMR spectra were acquired using a Bruker ASCEND 600 spectrometer (600 MHz; Sadis, Wissembourg, France).

### 4.2. Sample Materials

Unripe fruit from *Styrax japonica* Siebold et al. Zuccarini was collected from Ibaraki, Japan on 29 July 2013.

### 4.3. Isolation Procedure

After drying, the sample materials (5 g) were pulverised and extracted with methanol (50 mL) under two cycles of refluxing. The extract was then filtered, and the filtrate was evaporated to yield a syrup (1.16 g). A portion of the extract (500 mg) was suspended in water (50 mL) and then partitioned with *n*-hexane to remove the lipids. The aqueous layer was then further partitioned with ethyl acetate to extract low-polarity compounds, and the resulting aqueous layer was partitioned with *n*-butanol to extract glycoside compounds such as saponins. Finally, the remaining aqueous layer contained polar compounds, such as sugars and tannins. Subsequently, the *n*-butanol layer was evaporated, and the residue was subjected to column chromatography on silica gel (methanol-chloroform). The fractions (eluted with 20–50% methanol/chloroform) were combined and re-chromatographed using HPLC (column: Imtakt UK C-18, 20 mm × 250 mm, solvent: 80% methanol/water, flow rate: 8 mL/min; Portland, OR, USA) to obtain compounds **1** (27 mg) and **2** (6 mg).

The chemical structures of **1** and **2** were determined by spectroscopic techniques, including 2D-NMR (COSY, HMQC, and HMBC) and LC/MS. Using high-resolution MS, **1** and **2** were formulated as C_61_H_96_O_27_. The ^13^C-NMR spectra of compounds **1** and **2** were similar. Four moles of hexose signals and triterpene moiety signals were observed; moreover, the existence of tigloyl and acetyl groups was deduced. Therefore, compounds **1** and **2** were supposed to be analogs of jegosaponin. However, there were large differences in signals around C-21, 22, and 28 between **1** and **2**; **1** and **2** were deduced to be isomers of tigloyl and acetyl groups. The positions of the tigloyl and acetyl groups were determined using the HMBC spectrum. Finally, **1** and **2** were identified as jegosaponin A and B by comparison with the literature data [17]. Details of these compounds are described in Supplementary Materials.

### 4.4. Cell Viability Assay

Human prostate cancer PC-3, DU146, and LNCaP cells were obtained from Riken Bioresource Center (Tokyo, Japan) and cultured in Dulbecco’s modified Eagle medium-low glucose (DMEM; Sigma-Aldrich, St. Louis, MO, USA), supplemented with 10% fetal bovine serum (FBS; Biowest SAS, Nuaille, France) and 1% penicillin-streptomycin (Fujifilm Wako Pure Chemicals) at 37 °C in a humidified 5% CO_2_ atmosphere. For screening, the cells were seeded (3 × 10^3^ cells/well) in a 96-well plate and cultured for 24 h in DMEM supplemented with 1% FBS. The tested compounds were then administered to the cells. Forty-eight hours after administration, cell viability was measured using the CellTiter-Glo luminescent cell viability assay (Promega, Madison WI, USA) according to the manufacturer’s protocol. Luminescence signals were measured using a Victor2 microplate reader (PerkinElmer, Boston, MA, USA). For the negative control (0 μM jegosaponin A and B), 0.1% dimethyl sulfoxide (vehicle) was used. Cell viability was calculated as the ratio of the negative controls. The assay was performed in triplicate.

### 4.5. Zebrafish

Zebrafish AB strain (Zebrafish International Resource Center, Eugene, OR, USA) was maintained in our facility according to standard operational guidelines. All animal procedures were performed by the Ethics Committee of Mie University, according to the Japanese Animal Welfare Regulation ‘Act on Welfare and Management of Animals’ (Ministry of Environment of Japan), and complied with international guidelines.

### 4.6. Zebrafish Embryo Acute Toxicity Test (ZFET)

ZFETs were performed as previously reported [13]. In brief, blastula stage embryos (approximately 5 h post fertilisation (hpf)) were transferred into 6-well plates and exposed to jegosaponin A or B at 28 °C until 120 hpf. Each group contained nine fish in three triplicates. The number of surviving embryos was counted every 24 h.

### 4.7. Caspase 3/7 Assay

PC-3 cells were seeded (3 × 10^3^ cells/well) in a 96-well plate in DMEM containing 10% FBS for 24 h and treated with the test compounds. Caspase-Glo 3/7 assay (Promega) was performed according to the manufacturer’s instructions. Luminescence signals were measured using a Victor2 microplate reader (PerkinElmer).

### 4.8. Haemolysis Assay

A haemolysis assay was performed as previously reported [18]. Five millilitres of sheep defibrinated blood (Nippon Bio-Supp. Centre, Tokyo, Japan) was diluted in 45 mL of Dulbecco’s phosphate-buffered saline (DPBS) to obtain a total volume of 50 mL. The cell suspension was centrifuged at 500× *g* for 5 min, and the supernatant was discarded. This procedure was repeated three times, and DPBS was added to the precipitate, and the red blood cell (RBC) count was adjusted to ~5×10^8^ cells/mL. The RBC suspension (500 µL) was placed into a 1.5 mL storage tube, and the reagent was administered and incubated for 30 min on ice. Complete (100%) haemolysis was achieved with 1% Triton X-100 in RBC suspension. The cell suspension was then centrifuged at 500× *g* for 5 min. The haemoglobin concentration in the supernatant was measured by measuring the UV absorbance at 540 nm.

### 4.9. DRAQ7 Membrane Permeability Assay

PC-3 cells were seeded (4 × 10^3^ cells/well) in ibidi 8-well μ-Slide (Ibidi, Munich, Germany) in DMEM supplemented with 10% FBS and cultured for 24 h. Then, DRAQ7 (DRAQ7 DROP & GO; Biostatus, Shepshed, UK) and the tested compounds were added to the cells. After an appropriate time, the cells were imaged using a BZ-X710 fluorescence microscope (Keyence, Tokyo, Japan). For zebrafish embryos, 48 hpf embryos were treated with DRAQ7 and the tested compounds and imaged using a BZ-X710 fluorescent microscope.

### 4.10. Statistics

The data were statistically analysed using Student’s t-test or one-way analysis of variance with the Bonferroni-Dunn multiple comparison procedure, depending on the number of comparisons to be performed, using GraphPad Prism version 8 (GraphPad Software, San Diego, CA, USA). A p-value of less than 0.05, denoted the presence of a statistically significant difference between treatments.

## 5. Conclusions

We extracted cytotoxic jegosaponin A and jegosaponin B from unripe fruits of SJSZ, and characterised their strong detergent activities in vitro and in vivo. In addition to the traditional haemolysis assay, we also demonstrated their ability to disrupt cell membranes using a cell-impermeable fluorescent dye with cultured cells and zebrafish embryos. We consider that our new technologies are likely to assist other researchers in evaluating or analysing the cytotoxic mechanisms of other compounds.

## Figures and Tables

**Table 1:** Toxicity summary of jegosaponin A and B.

	RBC	PC-3 Cells		Zebrafish	
	Hemolysis HD_50_	LC_50_	DRAQ7 IC_50_	LC_50_	LC_50_
	(1 h)	(48 h)	(1 h)	(120 h)	(24 h)
Jegosaponin A	2.1 μM	0.7 μM	3.4 μM	0.5 μM	0.7 μM
Jegosaponin B	20.2 μM	1.6 μM	6.0 μM	1.3 μM	1.4 μM

## Data Availability

Not applicable.

## Supplementary Materials

The following are available online at https://www.mdpi.com/article/10.3390/ijms22126354/s1, Supplementary Methods: Details of the two compounds. Figure S1: Jegosaponin A and B suppress viability of human prostate cancer cells, Figure S2: Jegosaponin A and B do not affect caspase 3/7 activities in PC-3 cells. Jegosaponin A-1H-NMR, Jegosaponin A-13C-NMR, Jegosaponin B-1H-NMR, Jegosaponin B-13C-NMR.
