# Supplementary material for: Toxicity of Jegosaponins A and B from Styrax japonica Siebold et al. Zuccarini in Prostate Cancer Cells and Zebrafish Embryos Resulting from Increased Membrane Permeability
Source: Int J Mol Sci. 2021 Jun 14;22(12):6354. doi: 10.3390/ijms22126354 (PMC8246325; doi:10.3390/ijms22126354)
Supplement: Supplementary file 1 [file ijms-22-06354-s001.zip › ijms-1237207 210622_supple.pdf]

Supplementary Materials

Toxicity of jegosaponins A and B from Styrax japonica Siebold et al. Zuccarini in prostate cancer cells and zebrafish embryos resulting from increased membrane permeability

Supplementary Methods

Isolation procedure

Compound **1** (jegosaponin A): colourless amorphous, HR-ESI-MS (positive mode) *m/z* 1261.61790 $[M+H]^+$ (calcd. for $C_{61}H_{97}O_{27}$, 1261.6211), 1278.64359 $[M+NH_4]^+$ (calcd. for $C_{61}H_{100}O_{27}N$, 1278.6476). ^{13}C-NMR (C_5D_5N) d 38.9 (C-1), 26.6 (C-2), 89.9 (39.8), 39.8 (C-4), 55.8 (C-5), 18.6 (C-6), 33.3 (C-7), 40.2 (C-8), 47.0 (C-9), 36.9 (C-10), 24.0 (C-11), 124.2 (C-12), 143.0 (C-13), 41.9 (C-14), 34.8 (C-15), 68.2 (C-16), 48.1 (C-17), 40.2 (C-18), 47.4 (C-19), 36.6 (C-20), 79.6 (C-21), 74.5 (C-22), 28.1 (C-23), 16.9 (C-24), 15.8 (C-25), 17.0 (C-26), 27.6 (C-27), 63.9 (C-28), 29.7 (C-29), 20.3 (C-30), 21.0 (Ac), 171.2 (Ac-C̲O), 168.2 (Tig-1), 129.7 (Tig-2), 137.0 (Tig-3), 14.4 (Tig-4), 12.6 (Tig-5), 105.5 (GluA-1), 79.6 (GluA-2), 81.8 (GluA-3), 70.9 (GluA-4), 101.5 (Gal-1), 76.6 (Gal-2), 76.1 (Gal-3), 71.4 (Gal-4), 77.1 (Gal-5), 62.1 (Gal-6), 102.6 (Rha-1), 72.9 (Rha-2), 72.8 (Rha-3), 74.1 (Rha-4), 69.9 (Rha-5), 18.4 (Rha-6), 102.8 (Glc-1), 76.6 (Glc-2), 78.3 (Glc-3), 72.7 (Glc-4), 78.6 (Glc-5), 63.8 (Glc-6). Signals of GluA-5 and 6 were not detectable because of overlapping with noise. ^{1}H-NMR (C5D5N) d 6.60 (1H, d, *J*=9.9Hz, H-21), 6.28 (1H, d, *J*=9.9Hz, H-22), 6.25 (1H, d, *J*=7.9Hz, Gal-1), 5.97 (1H, d,*J*=7.8Hz, Glc-1), 5.36 (1H, m, H-12), 1.94 (3H, s, Tig-5), 1.88 (3H, s, Ac), 1.83 (3H, s, H-27), 1.63 (3H, d, *J*=7.2Hz, Tig-4), 1.32 (3H, s, H-30), 1.15 (3H, s, H-23), 1.09 (3H, s, H-29), 1.04 (3H, s, H-24), 0.80 (3H, s, H-26), 0.74 (3H, s, H-25).

Compound **2** (jegosaponin B): colourless amorphous, HR-ESI-MS (positive mode) *m/z* 1261.61793 $[M+H]^+$ (calcd. for $C_{61}H_{97}O_{27}$, 1261.6211), 1278.64472 $[M+NH_4]^+$ (calcd. for $C_{61}H_{100}O_{27}N$, 1278.6476). ^{13}C-NMR (C_5D_5N) d 38.9 (C-1), 26.6(C-2), 89.9 (C-3), 39.8 (C-4), 55.9 (C-5), 18.4 (C-6), 33.3 (C-7), 40.2 (C-8), 47.1 (C-9), 36.9 (C-10), 24.0 (C-11), 124.0 (C-12), 142.9 (C-13), 42.0 (C-14), 34.8 (C-15), 67.8 (C-16), 47.2 (C-17), 40.7 (C-18), 47.4 (C-19), 36.5 (C-20), 81.8 (C-21), 71.3 (C-22), 28.1 (C-23), 17.2 (C-24), 15.8 (C-25), 16.9 (C-26), 27.6 (C-27), 66.6 (C-28), 29.9 (C-29), 20.3 (C-30), 20.9 (Ac), 170.9 (Ac-C̲O), 168.7 (Tig-1), 130.0 (Tig-2), 135.9 (Tig-3), 14.3 (Tig-4), 12.6 (Tig-5), 105.5 (GluA-1), 79.5 (GluA-2), 82.7 (GluA-3), 71.4 (GluA-4), 77.6 (GluA-5), 172.4 (GluA-6), 101.5 (Gal-1), 76.5 (Gal-2), 76.2 (Gal-3), 71.4 (Gal-4), 77.2 (Gal-5), 62.1 (Gal-6), 102.6 (Rha-1), 72.8 (Rha-2), 72.8 (Rha-3), 74.0 (Rha-4), 70.0 (Rha-5), 18.6 (Rha-6), 102.8 (Glc-1), 76.6 (Glc-

2), 78.3 (Glc-3), 72.7 (Glc-4), 78.6 (Glc-5), 63.8 (Glc-6). ^{1}H-NMR (C5D5N) d 7.00 (1H, q, *J*=6.9Hz,Tig-3), 6.44 (1H, d, *J*=9.9Hz, H-21), 6.23 (1H. s, Rha-1), 6.19 (1H, d, *J*=7.8Hz, Gal-1), 5.90 (1H, d, *J*=7.5Hz, Glc-1), 5.43 (1H, m, H-12), 2.00 (3H, s, Ac), 1.84 (3H, s, Tig-5), 1.81 (3H, s, H-27), 1.58 (3H, d, *J*=7.0Hz, Tig-4), 1.40 (3H, d, *J*=6.1Hz, Rha-6), 1.30 (3H, s, H-30), 1.15 (3H, s, H-23), 1.09 (3H, s, H-29), 1.03 (3H, s, H-24), 0.96 (3H, s, H-26), 0.77 (3H, s, H-25).

Supplementary Figure

Figure S1

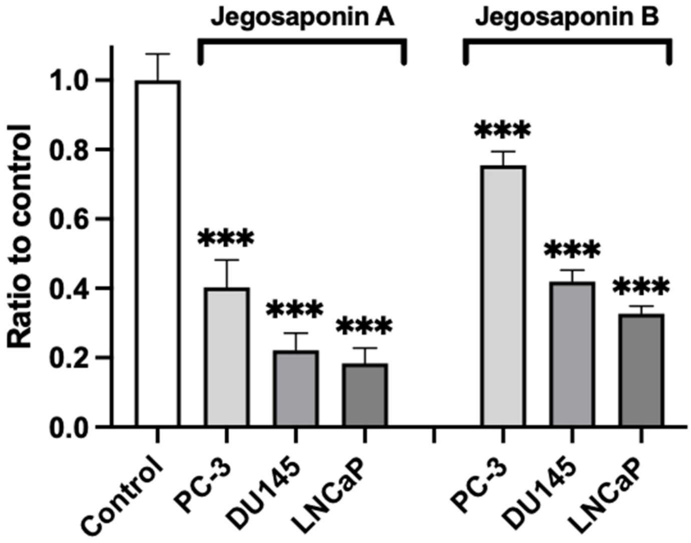

Figure S1. Jegosaponin A and B suppress viability of human prostate cancer cells. PC-3, DU145, and LNCaP cells were treated with 1 μM jegosaponin A or B for 48 h. n = 6, error bars indicate SD. ****p* < 0.001 vs. control.

Figure S2

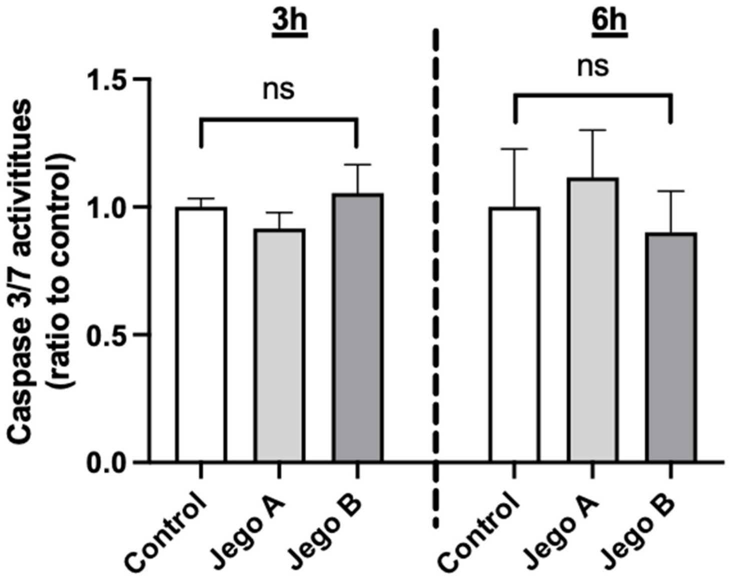

Figure S2. Jegosaponin A and B do not affect caspase 3/7 activities in PC-3 cells. PC-3 cells were treated with jegosaponin A (1.5 μM) or B (0.5 μM) for 3 or 6 h. These concentrations were at sub- LC_{50} concentrations (Table 1). n = 6, error bars indicate SD. ns indicates no significant difference.
