# Supplementary figures and images for: Toxicity of Jegosaponins A and B from Styrax japonica Siebold et al. Zuccarini in Prostate Cancer Cells and Zebrafish Embryos Resulting from Increased Membrane Permeability
Source: Int J Mol Sci. 2021 Jun 14;22(12):6354. doi: 10.3390/ijms22126354 (PMC8246325; doi:10.3390/ijms22126354)

20191119-Egonoki-10-4-1-py 13 1 C:\Bruker\TopSpin4.0.2\examdata

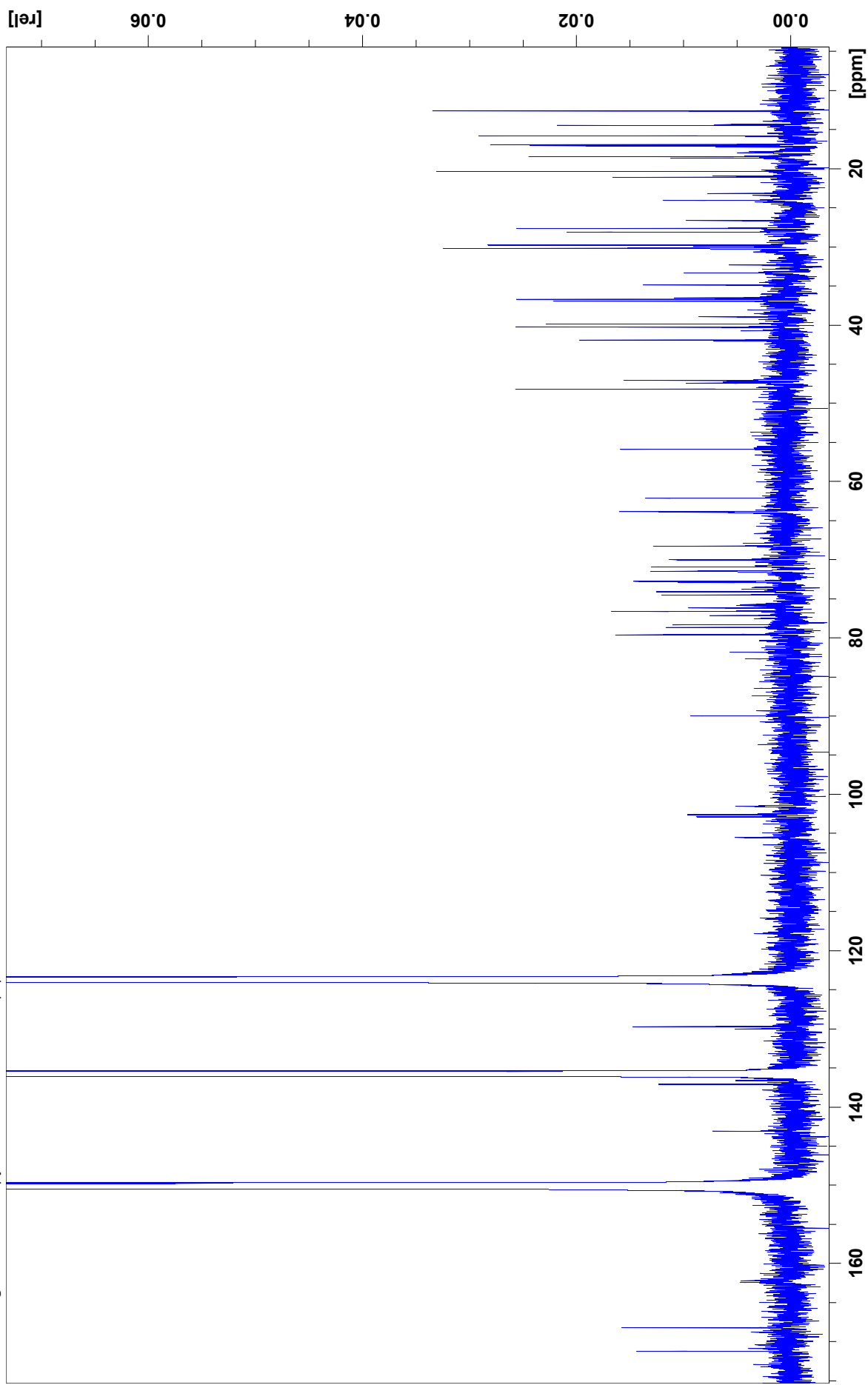

Supplement: Supplementary file 1 [file ijms-22-06354-s001.zip › ijms-1237207 jegosaponinA-13C-NMR-no-peak.pdf]

20191119-Egonoki-10-4-1-py 10 1 C:\Bruker\TopSpin4.0.2\examdata

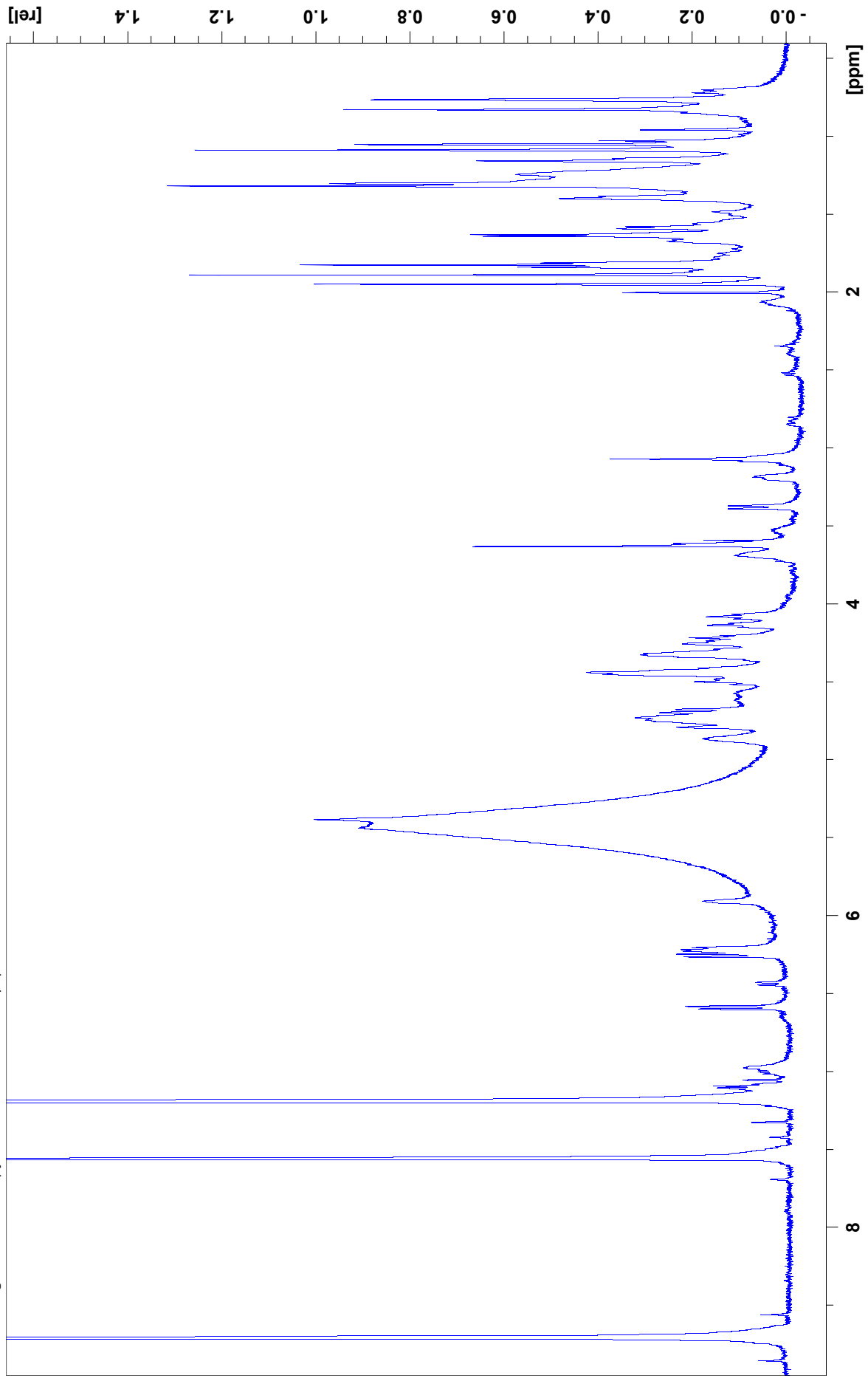

Supplement: Supplementary file 1 [file ijms-22-06354-s001.zip › ijms-1237207 jegosaponinA-1H-NMR-no-peak.pdf]

20200108-Egonoki-10-4-2-C5D5N 11 1 C:\Bruker\TopSpin4.0.2\examdata

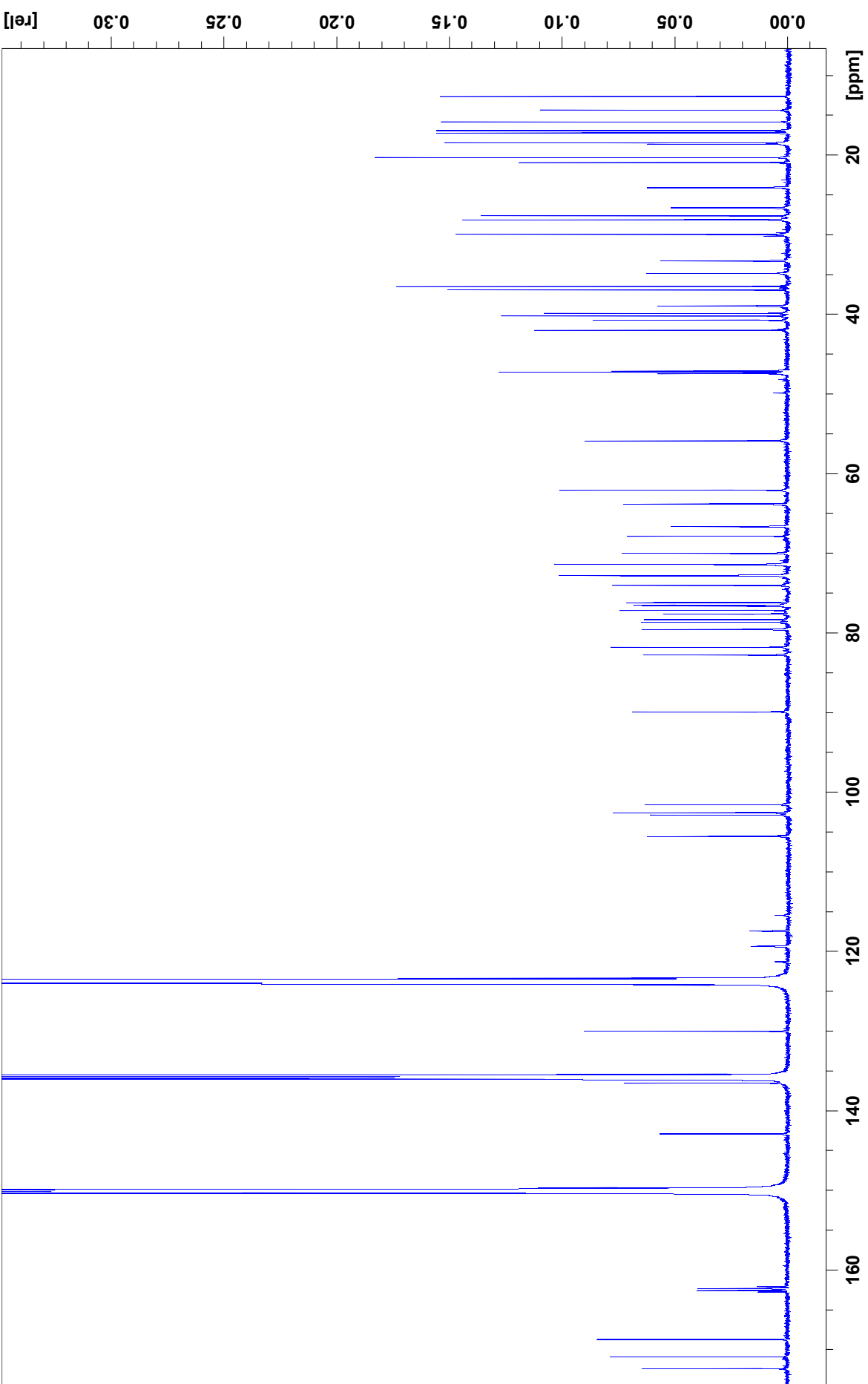

Supplement: Supplementary file 1 [file ijms-22-06354-s001.zip › ijms-1237207 jegosaponinB-13C-NMR-no-peak.pdf]

20200108-Egonoki-10-4-2-C5D5N 10 1 C:\Bruker\TopSpin4.0.2\data

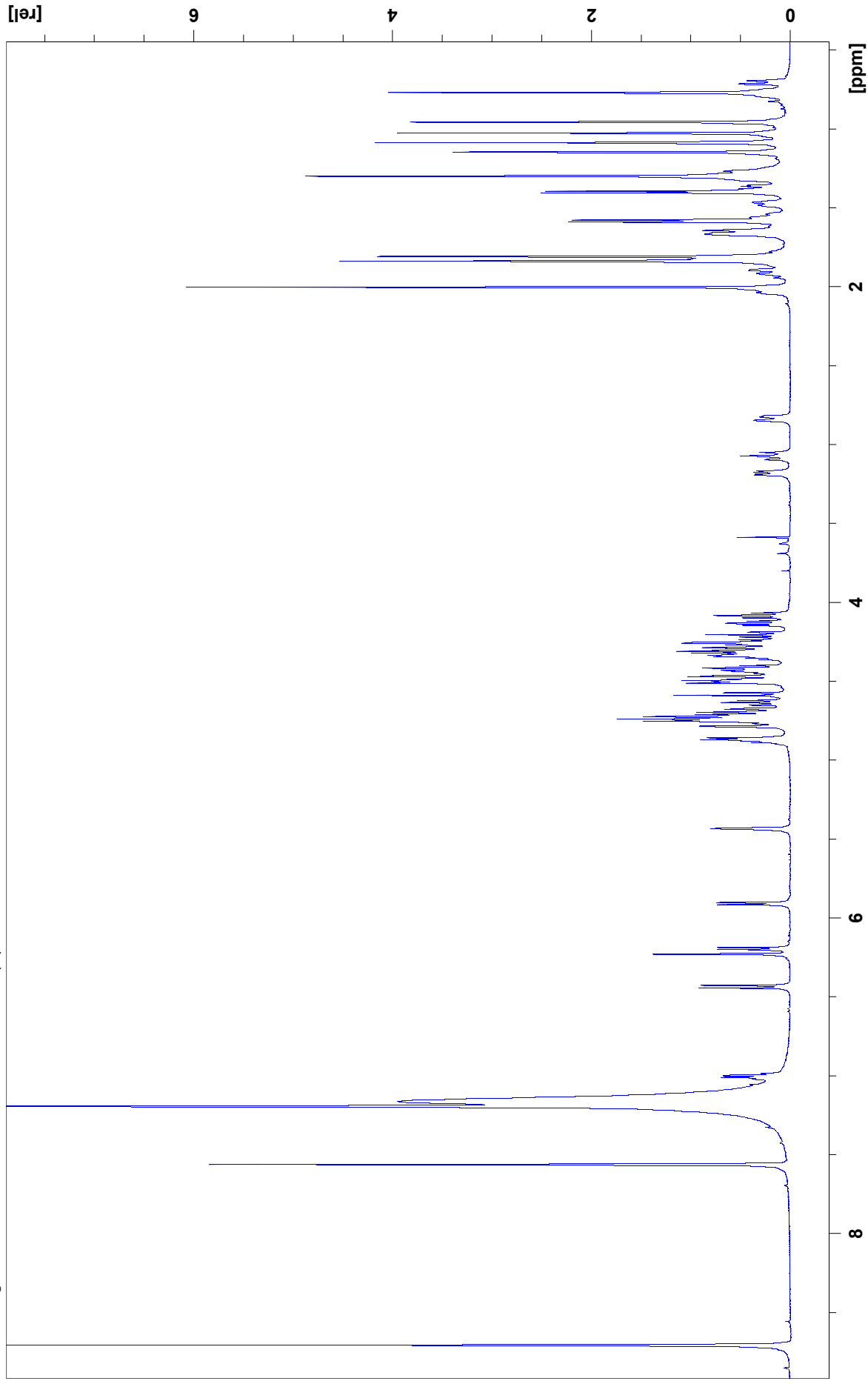

Supplement: Supplementary file 1 [file ijms-22-06354-s001.zip › ijms-1237207 jegosaponinB-1H-NMR-no-peak.pdf]
